# Crosstalk between YY1 and lncRNAs in cancer: A review

**DOI:** 10.1097/MD.0000000000031990

**Published:** 2022-12-09

**Authors:** Xiao-li Wang, Jing Li, Yan-hua Cao

**Affiliations:** a Department of Obstetrics and Gynecology, The Third Hospital of Xiamen, The First Affiliated Hospital of Xiamen University (Tongan Branch), Xiamen City, Fujian Province, China.

**Keywords:** cancer, lncRNAs, YY1

## Abstract

Transcription factor YY1 is an important regulator of many pathways in tumor cell growth, prognosis, epithelial-mesenchymal transition, invasion, and resistance to chemotherapy. These effects lead to upregulation of YY1 associated with poor outcomes in many tumors. Growing research evidence suggests that long non-coding RNAs (lncRNAs) play important roles in the regulatory network of YY1. YY1 can regulate lncRNA, and serve as the regulatory molecule of YY1, and lncRNA and YY1 even form a feedback loop. In this review, we summarize the relevant mechanisms of the interaction between YY1 and noncoding RNAs during tumor progression, which will provide a possible theoretical basis for the clinical treatment of tumors.

## 1. Introduction

The transcription factor, YY1, is a zinc finger DNA-binding protein in the Gli-Kruppel family, which can regulate gene expression according to chaperone protein, promoter status and chromatin structure.^[1]^ YY1 can interact with a variety of transcriptional regulators, including C-MYC, NOTCH, and YY1-related factors, and is involved in about 10% of the transcriptional control of mammalian genomes.^[1,2]^ YY1 can regulate transcription through replacement, functional interference, chromatin remodeling and induction of cofactor recruitment.^[3]^ YY1 has been proven to be abnormally expressed in many tumors and is associated with poor prognosis in patients. YY1 has been identified to be mainly involved in tumor cell growth, survival, epithelial-mesenchymal transformation, metastasis, and chemotherapy resistance.^[4]^ However, whether YY1 promotes or inhibits tumor growth is still controversial at this stage. For example, Zhang et al found that YY1 was highly expressed in pancreatic cancer compared with normal pancreatic tissues, and high overexpression of YY1 also predicted better survival outcomes in patients with pancreatic cancer. YY1 was also confirmed to inhibit the growth and metastasis of pancreatic cancer cells in vivo and in vitro.^[5]^ However, in melanoma, the increase of YY1 promotes melanoma metastasis, and YY1 can promote the proliferation, cell cycle progression, migration and invasion of melanoma cells.^[6]^ The molecular mechanism of the apparent contradictory effects of YY1 in tumors is still unclear, but it can be seen from the existing research results that this opposite regulatory state mainly depends on different tumor cell types.

**Figure 1.**
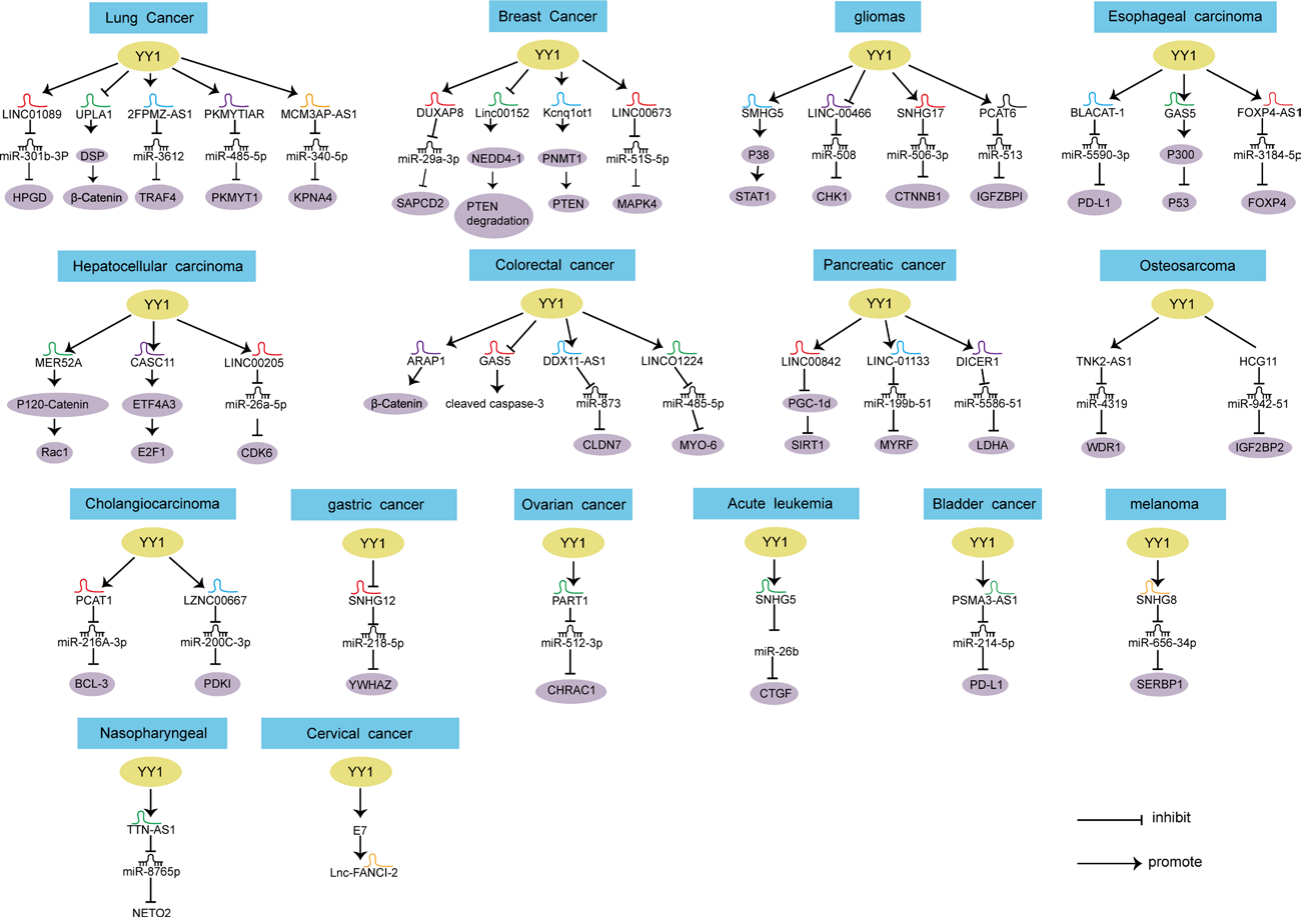
YY1 regulates the molecular mechanism of lncRNAs. lncRNAs = long non-coding RNAs.

With the rapid development of high-throughput technologies, we know from sequencing that protein-coding genes make up less than 2% of the total human genome, while most nucleotide sequences are transcribed by non-coding RNAs.^[7]^ Non-coding RNAs can also be divided into housekeeping RNAs (such as rRNA and tRNA) and regulatory RNAs (such as microRNAs, long non-coding RNAs [lncRNAs], and circRNAs). Non-coding RNAs with a length of more than 200 nucleotides are lncRNAs.^[8]^ lncRNAs are mainly involved in chromatin dynamics, gene expression, cell growth, differentiation and development, and transcriptome analysis results of sequencing showed that 1000 of lncRNAs showed abnormal expression or mutation in various tumors.^[9]^ lncRNAs play a key role in human tumors, but the specific regulatory mechanism of their involvement in tumors remains unclear. Recent studies have shown that lncRNAs can regulate YY1 network to control tumor growth. YY1 not only regulates the expression of lncRNAs (Fig 1), but also is an important downstream effector of lncRNAs. In this review, we mainly discuss the mechanism of crosstalk between YY1 and lncRNAs.

## 2. YY1 and lncRNAs

LncRNAs not only regulate chromatin remodeling and recruit transcription factors, but also may be involved in mRNA stability, translation, protein stability or localization. Recent studies have shown that YY1 has been confirmed to play a key role in lncRNAs regulatory network. In Table 1 and Table 2, we summarized the lncRNAs related to YY1 regulatory network.

**Table 1:** lncRNAs of YY1-regulated.

YY1-activated	Targeting	Cellular processes	References
ZFPM2-AS1	miR-3612	Cell proliferation, migration, and metastasis	14
MCM3AP-AS1	miR-340-5p	Cell proliferation, migration, and angiogenesis	15
PKMYT1AR	miR-485-5p	Cell proliferation, migration	17
Kcnq1ot1	DNMT1	Cell proliferation, migration, and metastasis	21
LINC00673	miR-515-5p	Cell cycle and apoptosis	23
DUXAP8	miR-29a-3p	Cell proliferation and apoptosis	24
SNHG5	p38	Cell proliferation	27
LINC00466	miR-508	Cell proliferation, migration, metastasis, and EMT	28
SNHG17	miR-506-3p	Cell proliferation and apoptosis	30
PCAT6	miR-513	Cell proliferation and apoptosis	31
ARAP1-AS1	*β*-catenin	Cell migration and EMT	34
DDX11-AS1	miR-873	Cell proliferation, migration, metastasis, and apoptosis	37
LINC01224	miR-485-5p	Cell proliferation, migration, metastasis, and apoptosis	38
MER52A	p120-catenin	Cell migration and metastasis	40
CASC11	EIF4A3	Cell proliferation, migration, apoptosis, and metabolic reprogramming	41
LINC00205	miR-26a-5p	Cell proliferation, cycle and apoptosis	42
LINC00842	PGC-1α	Cell proliferation and metastasis	44
DICER1-AS1	miR-5586-5p	Metabolic reprogramming	45
LINC01133	miR-199b-5p	Cell proliferation, migration, metastasis, and apoptosis	47
PART1	miR-512-3p	Cell proliferation, migration, metastasis, apoptosis, and drug resistance	49
lncRNA ESCCAL-1	RPLs	Cell proliferation	54
BLACAT1	miR-5590-3	Cell proliferation and metastasis	55
FOXP4-AS1	FOXP4	Cell proliferation	56
TTN-AS1	miR-876-5p/UPF1	Cell proliferation and metastasis	58
PCAT1	miR-216a-3p	Cell proliferation, migration, metastasis, and EMT	59
LINC00667	miR-200c-3p	Cell proliferation, migration, and metastasis	60
PSMA3-AS1	miR-214-5p	Cell proliferation, migration, metastasis, and apoptosis	62
SNHG8	miR-656-3p	Cell proliferation, migration, and metastasis	63
PCAT19	HNRNPAB	Cell proliferation, migration	64
SNHG5	miR-26b	Angiogenesis	65
TNK2-AS1	miR-4319	Cell proliferation, migration, and metastasis	66
YY1-repressed	Targeting	Cellular processes	References
lncRNA UPLA1	DSP	Cell proliferation, migration, metastasis, apoptosis, and cell cycle	13
LINC01089	miR-301b-3p	Cell proliferation, migration, and metastasis	18
LINC00152	PTEN	Cell proliferation and metastasis	20
GAS5	p300/cleaved PARP/caspase 3	cell proliferation, migration, metastasis, apoptosis, and cycle	35,57
SNHG12	miR-218-5p	Cell metastasis and EMT	61
HCG11	miR-942-5p/IGF2BP2	Cell proliferation	67

EMT = epithelial-mesenchymal transition, lncRNAs = long non-coding RNAs.

**Table 2:** lncRNAs of targeting YY1.

Targeting YY1	Regulation mode	Cellular processes	References
NPCCAT1	YY1 translation	Cell proliferation and migration	68
TLCD2-1	YY1 translation	Cell proliferation and drug resistance	69
LINC00899	YY1 transcription	Cell proliferation and apoptosis	70
HOTAIR	YY1 translation	Cell proliferation, migration, metastasis, and apoptosis	71
GACAT1	YY1 translation	Cell proliferation, apoptosis, and cell cycle	72
LINC00958	YY1 transcription	Cell proliferation	73
CASC15	YY1 activity	Cell proliferation	75
LINC00668	YY1 activity	Cell proliferation, migration, metastasis and EMT	76
TCONS_00012883	YY1 activity	Cell proliferation and metastasis	77
LINC00278	YY1 activity	Cell apoptosis	78
USP21	YY1 protein stability	Cell proliferation, migration, and metastasis	79
SNHG14	YY1 activity	Cell proliferation, metastasis, and apoptosis	80

EMT = epithelial-mesenchymal transition, lncRNAs = long non-coding RNAs.

### 2.1. YY1-regulated lncRNAs

#### 2.1.1. *YY1-regulated lncRNAs and lung cancer*.

Lung cancer is one of the most common cancers in the world, with nearly 2 million new cases every year, so it is important to further explore the mechanisms involved in lung cancer.^[10]^ YY1-regulated lncRNAs promote tumor progression through multiple mechanisms. lncRNA PVT1 is involved in the progression of various tumors, including non-small cell lung cancer, hepatocellular carcinoma, gastric cancer, breast cancer and glioma.^[11]^ Researchers found that YY1 can directly bind to the promoter region of PVT1 to promote tumor growth in lung cancer.^[12]^ Similar studies have shown that UPLA1 is associated with the progression and prognosis of lung cancer. It was determined that YY1 could bind to UPLA1 at sites 1: 280 to 291, 2: 633 to 644, 3: 636 to 641 and 4: 912 to 923, and reverse regulate the expression of lncRNA-UPLA1.^[13]^ miRNA also participates in YY1-regulated lncRNAs network in lung cancer. YY1-mediated ZFPM2-AS1 promotes down-regulation of miR-3612 activity, while TNF receptor-associated factor 4 (TRAF4) interacts with miR-3612 in lung cancer.^[14]^ YY1-induced MCM3AP-AS1 promotes cell proliferation, migration and angiogenesis in lung cancer cells by targeting miR-340-5p/Karyopherin *α* 4 axis. Further rescue tests showed that Karyopherin α 4 overexpression attenuates the inhibitory effect of MCM3AP-AS1 silencing on angiogenesis and progression of lung cancer cells.^[15]^ PKMYT1 is a major player in cell cycle regulation and DNA damage recognition and repair in cancer cells.^[16]^ PKMYT1AR induced by YY1 directly interacts with miR-485-5p to inhibit the expression of oncogene PKMYT1. PKMYT1 promotes the dryness of cancer stem cells through *β*-catenin ubiquitin degradation, leading to lung cancer progression.^[17]^ In additional, LINC01089 inhibited by YY1 competes with endogenous RNA of miR-301b-3p to promote hydroxyprostaglandin dehydrogenase expression.^[18]^

#### 2.1.2. *YY1-regulated lncRNAs and breast cancer*.

Breast cancer is one of the most common cancers in women, and the number of new cases of cancer continues to rise gradually each year.^[19]^ Studies have shown that binding YY1 to LINC00152 promoter blocks LINC00152 transcription. The up-regulated LINC00152 significantly promoted the ubiquitination and degradation of PTEN protein in breast cancer.^[20]^ YY1 promote the transcription of Kcnq1to1, which mediated PTEN methylation through DNMT1, thereby inhibiting the expression of PTEN in triple negative breast cancer.^[21]^ PTEN is a tumor suppressor gene in breast cancer and is frequently mutated/lost in tumors.^[22]^ miRNA also participates in YY1-regulated lncRNAs network in breast cancer. YY1 binds to LINC00673 promoter to increase its cis-transcription. LINC00673 promotes tumor cell growth by regulating breast cancer cell cycle and apoptosis. This result was achieved mainly by sponging miR-515-5p to act as ceRNA to regulate MARK4 (Microtubule affinity regulating kinase 4) expression and thereby inhibit Hippo signaling pathway.^[23]^ DUXAP8 acts as a miR-29a-3p sponge to promote the expression of the downstream gene SAPCD2 (Suppressor APC Domain Containing 2) in breast cancer. YY1 promoted the transcriptional activation of DUXAP8.^[24]^

#### 2.1.3. *YY1-regulated lncRNAs and gliomas*.

Glioma is the most common primary tumor of the central nervous system, and about half of gliomas are diagnosed as glioblastoma, a highly aggressive brain tumor.^[25]^ Aging is an important factor associated with poor prognosis of glioblastoma, and YY1 binds to the promoter domain of TP73-AS1 in the aging brain to promote its transcription.^[26]^ YY1 promoted the transcription of SNHG5 in glioblastoma, and it was found that SNHG5 could enhance the carcinogenic role of P38/MAPK signaling pathway in glioblastoma.^[27]^ miRNA also participates in YY1-regulated lncRNAs network in gliomas. LINC00466 is up-regulated and is associated with poor prognosis in gliomas. Silencing LINC00466 inhibited the growth and metastasis of tumor cells and promoted apoptosis. Mechanistically, YY1 can directly bind to the promoter region of LINC00466, which can bind to miR-508 to inhibit its expression and act as an endogenous sponge to regulate the expression of CHK1 (checkpoint kinase I).^[28]^ CHK1 can participate in cell DNA replication, mitosis process and DNA repair.^[29]^ YY1-promoting transcriptional SNHG17 interacts with miR-506-3p to promote the expression of CTNNB1(Catenin *β*1), which is an important protein in activating Wnt/*β*-catenin signaling pathway in gliomas.^[30]^ YY1 promoted up-regulation of PCAT6 in glioblastoma, and PCAT6 promoted downstream insulin-like growth factor-2 mRNA-binding protein 1 (IGF2BP1) protein expression in a competitive endogenous RNA manner through miR-513.^[31]^ IGF2BP1 is involved in tumor cell proliferation, metastasis and chemical resistance.^[32]^

#### 2.1.4. *YY1-regulated lncRNAs and colorectal cancer*.

Colorectal cancer remains one of the most common cancers with a global disease burden, with one of the highest morbidity and mortality rates among cancers.^[33]^ luciferase reporter gene detection showed that YY1 had strong activity on the ARAP1-AS1 promoter. YY1 promoted the expression of ARAP1-AS1, and promoted the proliferation and metastasis of tumor cells through Wnt/*β*-catenin signaling pathway.^[34]^ The study confirmed that the variation of GAS5 promoter RS55829688 was related to the risk of colorectal cancer, and the combination of YY1 and GAS5 negatively regulated the expression of GAS5.^[35]^ GAS5 inhibits the proliferation and metastasis of colorectal cancer by regulating the degradation of YAP phosphorylation, and is negatively regulated by m6A reader YTHDF3.^[36]^ Further studies found that there was also a regulatory relationship between the YY1-regulated lncRNAs network and miRNA in colorectal cancer. DDX11-AS1 promoted by YY1 promotes tumor cell proliferation, migration and invasion and inhibits apoptosis by targeting the miR-873/CLDN7 axis.^[37]^ In addition, YY1 promoted the proliferation and metastasis and increased the inhibition of apoptosis through LINC01224/miR-485-5p/MYO-6 axis.^[38]^

#### 2.1.5. *YY1-regulated lncRNAs and hepatocellular carcinoma*.

Hepatocellular carcinoma is a high incidence tumor in the world. Despite the continuous improvement of medical technology in recent years, the mortality rate of hepatocellular carcinoma is still increasing due to the fact that most of the patients detected are in advanced stage.^[39]^ YY1 binds to the MER52A promoter and promotes transcription. The MER52A promotes hepatocellular carcinoma cell migration and invasion by stabilizing p120-catenin and activating the p120-catenin/Rac1/Cdc42 axis.^[40]^ YY1 directly binds to the promoter of CASC11, and YY1 knockdown inhibits the expression of CASC11. CASC11 promotes hepatocellular carcinoma growth through EIF4A3 mediated E2F1.^[41]^ Up-regulated LINC00205 is positively correlated with grade and leads to poor prognosis of hepatocellular carcinoma, and LINC00205 knockout promotes cell cycle arrest and apoptosis of tumor cells in *G*0/*G*1 phase. Further study of the mechanism found that LINC00205 induced by YY1 directly binds to miR-26a-5p to promote CDK6 expression and promote the progression of HCC cells.^[42]^

#### 2.1.6. *YY1-regulated lncRNAs and pancreatic cancer*.

Pancreatic cancer has become the third leading cause of tumor-related death in the United States, and new cases and deaths continue to increase each year.^[43]^ LINC00842 participates in metabolic remodeling in pancreatic cancer cells and is deacetylated by SIRT1 (Sirtuin 1) by inhibiting the acetylation of Peroxisome proliferator-activated receptor-*γ* coactivator. Upstream mechanism studies have shown that exposure to YY1 in high glucose levels promotes the expression of LINC00842 in tumor cells.^[44]^ YY1 binds to the DICER1-AS1 promoter to promote its transcription, and the up-regulated DICER1-AS1 acts as a sponge for miR-5586-5p and inhibits the expression of glycolysis genes, including LDHA, HK2, PGK1 and SLC2A1.^[45]^ LINC01133 regulates a variety of malignancies, including cancers of the digestive system, female reproductive system, respiratory system and urinary system.^[46]^ LINC01133 can recruit YY1 on its promoter. Upregulated LINC01133 directly interacts with miR-199b-5p to target the myelin regulatory factor, thereby promoting pancreatic cancer growth and metastasis.^[47]^

#### 2.1.7. *YY1-regulated lncRNAs and gynecologic related tumor*.

Ovarian cancer is usually detected in patients at an advanced stage and is the most common cause of gynecological cancer death, which is often associated with recurrence. It is therefore important to identify specific mechanisms for the progression of ovarian cancer.^[48]^ PART1 can promote the proliferation and metastasis of ovarian cancer cells, which can improve the potential targets for the treatment of ovarian cancer. YY1-induced PART1 directly binds to the miR-512-3p target and regulates the CHRAC1 (Chromatin Accessibility Complex Subunit 1), thereby increasing the cisplatin resistance of ovarian cancer.^[49]^ Cervical cancer is a common gynecological tumor and is closely related to high risk subtypes of human papillomavirus (HPV).^[50]^ High-risk-HPV infection is an important factor causing cervical cancer in women. lnc-FANCI-2 is transcribed and selectively spliced by 2 alternative promoters and polyadenylated at 1 of 2 alternative poly (A) sites. YY1 can interact with E7 CR3 core gene to trans-activate the promoter of lnc-FANCI-2. In addition, HPV18 promoted YY1 expression by reducing the 3’ untranslated region of miR-29a targeted YY1 mRNA.^[51]^ Endometriosis has been considered as a precursor to neoplasms associated with endometriosis.^[52]^ In endometriosis, the PROMO and AnimalTFDB databases predict that lncRNAs (MALAT1, NEAT1, SNHG22, and XIST) may interact with YY1.^[53]^

#### 2.1.8. *YY1-regulated lncRNAs and other malignant tumors*.

With the confirmation of YY1 and lncRNAs as tumor regulatory networks, more and more researchers have conducted further studies on their crosstalk mechanism and found that YY1-regulated lncRNAs are involved in the regulation of esophageal squamous cell carcinoma, laryngeal squamous cell carcinoma, nasopharyngeal carcinoma, cholangiocarcinoma, gastric cancer, bladder cancer, melanoma, prostate cancer, acute leukemia and osteosarcoma. in esophageal squamous cell carcinoma, YY1 has been confirmed to bind to the hypomethylation promoter region of ESCCAL-1,^[54]^ and YY1-induced BLACAT1 was able to compete with PD-L1 for binding to miR-5590-3p in esophageal cancer cells.^[55]^ FOXP4-AS1 can up-regulate FOXP4 expression and promote the proliferation of esophageal squamous cell carcinoma by sponging miR-3184-5p, and YY1 can promote the activation transcription of FOXP4-AS1.^[56]^ YY1 promotes the progress of laryngeal squamous cell carcinoma and telomerase activity by inhibiting the stability of GAS5-dependent p53 or reducing the stability of p53 by binding to P300.^[57]^ Chen et al found that TTN-AS1 activated by YY1 transcription interacts with miR-876-5p and UPF1 to promote the expression of NETO2, thereby promoting the proliferation and metastasis of nasopharyngeal carcinoma.^[58]^ As an oncogene of cholangiocarcinoma, PCAT1 can be activated and transcribed by YY1. PCAT1 mainly exists in the cytoplasm and promotes the expression of BCL3 through the PCAT1/miR-216A-3p axis.^[59]^ In addition, YY1 promotes transcriptional activation of LINC00667, and then LINC00667 promotes the expression of PDK1 by binding to miR-200c-3p to promote the progress of cholangiocarcinoma.^[60]^ SNHG12 negatively regulated by YY1 promotes the progression of gastric cancer through the miR-218-5p/YWHAZ axis and the stabilization of CTNNB1 protein by promoting the activity of *β*-catenin.^[61]^ Zhang et al found that YY1 promotes the transcriptional activation of PSMA3-AS1, and targets miR-214-5p to promote the expression of PD-L1 in bladder cancer cells.^[62]^ YY1 can also promote the transcriptional activity of SNHG8, and then directly interact with miR-656-3p to regulate SERPINE1 mRNA binding protein 1 (SERBP1) in melanoma.^[63]^ Hua et al ‘s study in prostate cancer showed that the combination of RS 1,16,72,691 and RS 8,87,391 with promoters of transcription factors NKX3.1 and YY1 regulates the expression of short isoform of PCAT19 by regulating the binding of transcriptional activators YY1 to their promoters. The risk variants of RS 1,16,72,691 and RS 8,87,391 attenuated the binding of transcription factors YY1 to the short isoform of PCAT19 promoter, leading to reduced promoter activity but enhanced promoter activity, followed by activation of long isoform of PCAT19.^[64]^ YY1 promotes transcription of SNHG5 and regulates angiogenesis of acute leukemia by targeting miR-26b to activate CTGF/VEGFA.^[65]^ YY1 promotes the transcription of TNK2-AS1, thereby promoting osteosarcoma progression through TNK2-AS1/miR-4319/WDR1 axis.^[66]^ YY1 inhibits transcriptional activation of HCG11 by binding to the promoter region of HCG11. HCG11 interacts with downstream molecules miR-942-5p and IGF2BP2 to promote the expression of P27 Kip1, which is an important molecule in the biological function of osteosarcoma.^[67]^

### 2.2. LncRNAs regulating YY1

#### 2.2.1. *LncRNAs controlling YY1 transcription or translation*.

**Figure 2.**
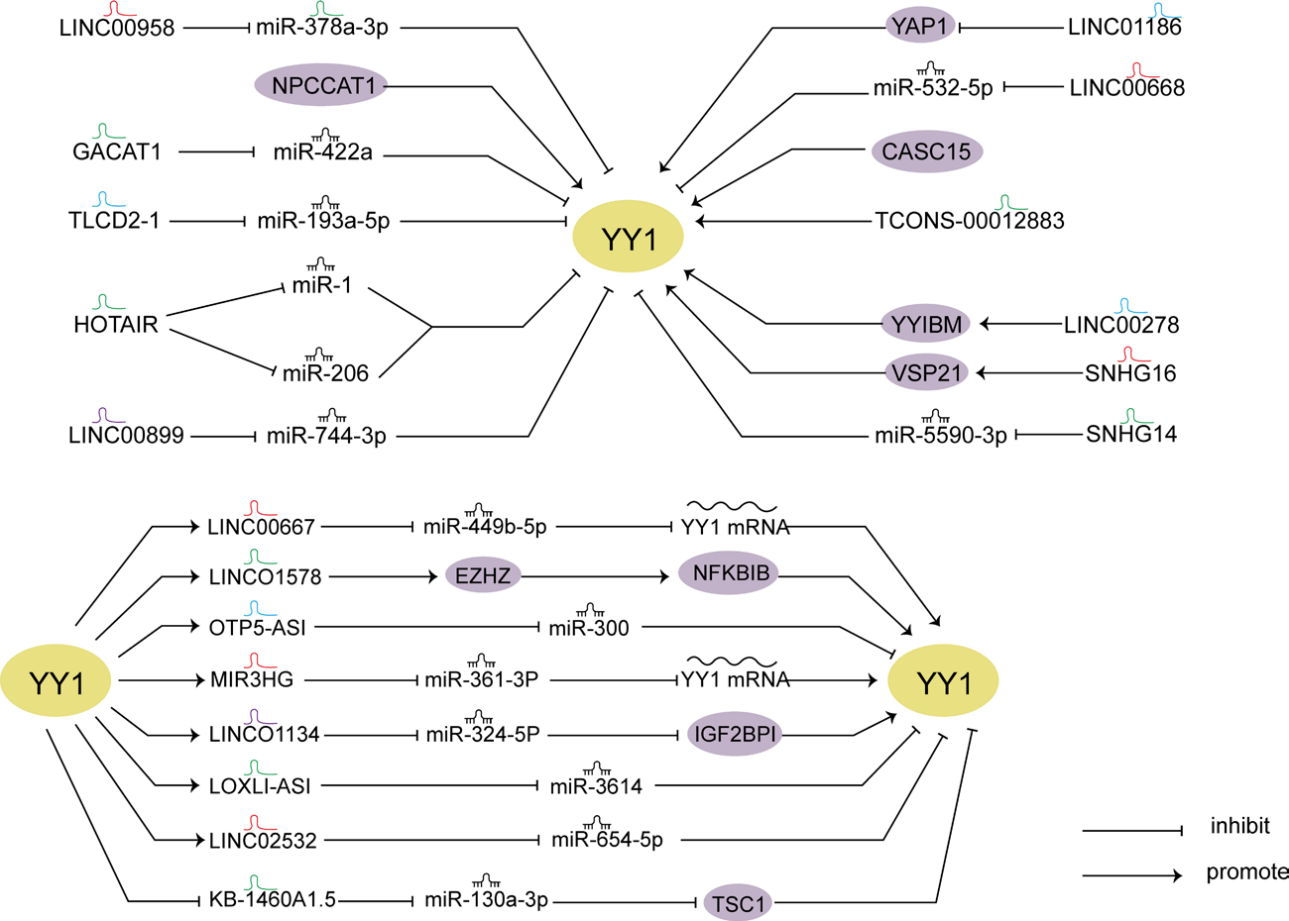
Crosstalk between YY1 and lncRNAs in cancer. (A) lncRNA regulates YY1 networks. (B) Feedback loops between YY1 and lncRNAs. lncRNAs = long non-coding RNAs.

In nasopharyngeal carcinoma, some scholars have found that NPCCAT1 directly binds to YY1 mRNA 5’UTR, promotes YY1 mRNA translation. The results of cell biological function showed that YY1 promoted the proliferation and migration of tumor cells. In addition, the results of salvage experiments showed that YY1 knockout on the basis of NPCCAT1 overexpression weakened the effect of NPCCAT1 in promoting tumor growth.^[68]^ In addition, upregulation of TLCD2-1 leads to radiation resistance in colorectal cancer. TLCD2-1 promotes the expression of YY1 mRNA by targeting miR-193A-5p, thus regulating the colorectal cancer immune infiltrating microenvironment.^[69]^ LINC00899 mainly exists in the cytoplasm and is highly expressed, which is associated with poor prognosis in patients. Animal experiments show that LINC00899 promotes tumor growth. miR-744-3p is the downstream gene of LINC00899, and miR-744-3p directly interacts with YY1 to regulate the mRNA expression of YY1.^[70]^ In medulloblastoma, HOTAIR acts as a sponge of miR-1 and miR-206 to promote YY1 mRNA and protein expression. Cell experiments revealed that HOTAIR promoted tumor growth and metastasis of tumor cells and inhibited apoptosis by miR-1/miR-206-YY1 axis.^[71]^ GACAT1 binds to miR-422a to regulate the expression of YY1 mRNA and protein in NSCLC cells.^[72]^ Finally, the research found that LINC00958 is associated with poor prognosis of breast cancer. m6A-induced LINC00958 acts as a spongiform for miR-378a-3p to target YY1 and promote the progression of breast cancer.^[73]^ These results suggest that lncRNA controls YY1 mRNA stability or translation (Fig 2A).

#### 2.2.2. *LncRNAs affecting YY1 protein stability or activity*.

As shown in Figure 2A, lncRNA affects YY1 protein stability or activity (Fig 2A). LINC01186 is overexpressed in thyroid papillary carcinoma and blocks tumor suppressor kinase 1 signal transduction by inhibiting the expression of YY1-related proteins.^[74]^ CASC15 promotes YY1-mediated transcriptional regulation of SOX4 promoter and enhances downstream effects of SOX4.^[75]^ In hepatocellular carcinoma, LINC00668 promotes YY1 protein expression by miR-532-5p, thereby accelerating cell proliferation, metastasis, and epithelial-mesenchymal transition processes.^[76]^ TCONS_00012883 is mainly located in the nucleus and interacts with DDX3 to up-regulate target gene MMP1 and promote colorectal cancer progression. Knocking TCONS_00012883 inhibited the enrichment of YY1 on the MMP1 promoter, while lncRNA-TCONS_00012883 overexpression enhanced the enrichment.^[77]^ Wu et al found that LINC00278 is a novel Y-linked lncRNA capable of encoding YY1-binding micropeptide (YY1BM). YY1BM blocks the interaction between YY1 and androgen receptor, thereby inhibiting the expression of eEF2K, while smoking reduces the m6A modification of lLINC00278 and YY1BM translation in esophageal squamous Cell Carcinoma.^[78]^ In small-cell lung cancer, YY1 promotes transcriptional activation of SNHG16, which promotes USP21 expression through miR-4500, and USP21 stabilizes protein levels by deubiquitination of YY1.^[79]^ Another similar study found that SNHG14 silencing can inhibit the protein expression of Cyclin *D*1 and N-cadherin, and promote the expression of Bax, Cleaved caspase-3 and e-cadherin. Further study of the mechanism found that SNHG14 directly binds to miR-5590-3p, while the 3’UTR of miR-5590-3p can interact with YY1 protein in prostate cancer.^[80]^

### 2.3. Feedback loops between YY1 and lncRNAs

Some lncRNAs induced by YY1 can also conversely regulate the expression level of YY1 (Fig 2B). Yu et al found that YY1 promoted transcriptional activation of LINC00667 and further regulated the progression of colorectal cancer through downstream target miR-449B-5p. Further mechanism studies found that YY1 overexpression weakened the promoting effect of LINC00667 on colorectal cancer cell growth, and LINC00667 promoted the expression of YY1 mRNA and protein through miR-449b-5p.^[81]^ LINC01578 was shown to be a chromatin binding lncRNA. The study has confirmed that LINC01578 is associated with metastasis and poor prognosis of colorectal cancer, and functional experiments have shown that LINC01578 can promote liver metastasis of colon cancer. Mechanistically, YY1 binds to the promoter of LINC01578 to promote its transcriptional activity, and LINC01578 interacts with EZH2 to recruit it to the NFKBIB promoter, thereby activating NF-*κ*B signal transduction. However, LINC01578 in turn promotes its own promoter activity by activating the NF-*κ*B/YY1 axis.^[82]^ Mechanistically, activation by binding to OIP5-AS1 promoter, and OIP5-AS1 directly targets miR-300. Further studies found that miR-300 targets YY1 to regulate the WNT pathway. The results revealed that OIP5-AS1/miR-300/YY1 axis formed a feedback loop.^[83]^ Up-regulated MIR31HG promotes glycolysis of colorectal cancer, vascular endothelial cell formation, and lung metastasis. Mechanistically, up-regulation of YY1 can promote the transcriptional activity of MIR31HG, and overexpression of MIR31HG can further promote the expression of YY1.^[84]^

LINC01134 knockdown inhibits hepatocellular carcinoma proliferation, metastasis and promotes apoptosis, and inhibits tumor growth in vivo. Further mechanism studies found that YY1 promotes transcription by binding to promoters at sites -773 to -762 upstream of LINC 01134 transcription termination site. LINC01134 acts as a miR-324-5p sponge and interacts with IGF2BP1 to increase the stability of YY1 mRNA expression. Finally, overexpressed YY1 can promote the transcription of LINC01134. The results show that there is a feedback loop between YY1 and LINC01134.^[85]^ LOXL1-AS1 interacts with miR-3614-5p and binds to the target gene YY1 in hepatocellular carcinoma. Further study of the mechanism found that YY1 also promoted the transcriptional activity of LOXL1-AS1.^[86]^ Silencing LINC02532 enhances radiosensitivity of clear cell renal cell carcinoma by inhibiting DNA double-strand breaks repair. Mechanistically, on the 1 hand, YY1 promotes its transcription by binding to LINC02532 promoter, and on the other hand, LINC02532 interacts with miR-654-5p to promote YY1 expression.^[87]^ KB-1460A1.5 can affect the metabolism of amino acids in glioma. The KB-1460A1.5/miR-130a-3p axis regulates the expression of the transcription factor YY1 through the key gene TSC1 of the mTOR pathway, and the expressed YY1 directly binds to the kB-1460A1.5 promoter to feedback and regulate the transcription of KB-1460A1.5.^[88]^

## 3. Conclusions

Many studies have confirmed that YY1 and lncRNAs have close and complex crosstalk, which play a crucial role in tumor progression. YY1 can regulate lncRNAs, and lncRNAs can also serve as the regulatory molecule of YY1, and lncRNAs and YY1 even form a feedback loop. Although we have described the mechanisms related to YY1 and lncRNA network in recent years, the mechanisms still need to be further discussed before we can have a comprehensive and clear understanding of malignant tumor transformation. The research of YY1 in tumor has become more and more mature, but the specificity of its drug is still poor. In principle, loss or recovery of lncRNAs in the YY1 network could be an alternative to YY1 drugs. Therefore, we urgently need to systematically elucidate the mechanism of mutual regulation between YY1 and non-coding RNAs, which will provide a possible theoretical basis for the clinical treatment of tumors.

## Author contributions

**Conceptualization:** Xiao-li Wang, Jing Li, Yan-hua Cao.

**Data curation:** Yan-hua Cao.

**Writing – original draft:** Xiao-li Wang, Yan-hua Cao.

**Writing – review & editing:** Xiao-li Wang, Yan-hua Cao.

## References

[1] Khachigian LM. The Yin and Yang of YY1 in tumor growth and suppression. Int J Cancer. 2018;143:460–5. 29322514 10.1002/ijc.31255

[2] Schug J Schuller WP Kappen C. Promoter features related to tissue specificity as measured by Shannon entropy. Genome Biol. 2005;6:R33. 15833120 10.1186/gb-2005-6-4-r33 PMC1088961

[3] Gordon S Akopyan G Garban H. Transcription factor YY1: structure, function, and therapeutic implications in cancer biology. Oncogene. 2006;25:1125–42. 16314846 10.1038/sj.onc.1209080

[4] Hays E Bonavida B. YY1 regulates cancer cell immune resistance by modulating PD-L1 expression. Drug Resist Updat. 2019;43:10–28. 31005030 10.1016/j.drup.2019.04.001

[5] Zhang JJ Zhu Y Xie KL. Yin Yang-1 suppresses invasion and metastasis of pancreatic ductal adenocarcinoma by downregulating MMP10 in a MUC4/ErbB2/p38/MEF2C-dependent mechanism. Mol Cancer. 2014;13:130. 24884523 10.1186/1476-4598-13-130 PMC4047260

[6] Zhao G Li Q Wang A. YY1 regulates melanoma tumorigenesis through a miR-9 ~ RYBP axis. J Exp Clin Cancer Res. 2015;34:66. 26104682 10.1186/s13046-015-0177-y PMC4511530

[7] Djebali S Davis CA Merkel A. Landscape of transcription in human cells. Nature. 2012;489:101–8. 22955620 10.1038/nature11233 PMC3684276

[8] Tu R Chen Z Bao Q. Crosstalk between oncogenic MYC and noncoding RNAs in cancer. Semin Cancer Biol. 2021;75:62–71. 33160022 10.1016/j.semcancer.2020.10.014

[9] Bhan A Soleimani M Mandal SS. Long noncoding RNA and cancer: a new paradigm. Cancer Res. 2017;77:3965–81. 28701486 10.1158/0008-5472.CAN-16-2634 PMC8330958

[10] Thai AA Solomon BJ Sequist LV. Lung cancer. Lancet. 2021;398:535–54. 34273294 10.1016/S0140-6736(21)00312-3

[11] Pan X Zheng G Gao C. LncRNA PVT1: a novel therapeutic target for cancers. Clin Lab. 2018;64:655–62. 29739059 10.7754/Clin.Lab.2018.171216

[12] Huang T Wang G Yang L. Transcription factor YY1 modulates lung cancer progression by activating lncRNA-PVT1. DNA Cell Biol. 2017;36:947–58. 28972861 10.1089/dna.2017.3857

[13] Han X Jiang H Qi J. Novel lncRNA UPLA1 mediates tumorigenesis and prognosis in lung adenocarcinoma. Cell Death Dis. 2020;11:999. 33221813 10.1038/s41419-020-03198-y PMC7680460

[14] Yan Z Yang Q Xue M. YY1-induced lncRNA ZFPM2-AS1 facilitates cell proliferation and invasion in small cell lung cancer via upregulating of TRAF4. Cancer Cell Int. 2020;20:108. 32280300 10.1186/s12935-020-1157-7 PMC7126398

[15] Li X Yu M Yang C. YY1-mediated overexpression of long noncoding RNA MCM3AP-AS1 accelerates angiogenesis and progression in lung cancer by targeting miR-340-5p/KPNA4 axis. J Cell Biochem. 2020;121:2258–67. 31693222 10.1002/jcb.29448

[16] Ghelli Luserna di Rorà A Cerchione C Martinelli G. A WEE1 family business: regulation of mitosis, cancer progression, and therapeutic target. J Hematol Oncol. 2020;13:126. 32958072 10.1186/s13045-020-00959-2 PMC7507691

[17] He Y Jiang X Duan L. LncRNA PKMYT1AR promotes cancer stem cell maintenance in non-small cell lung cancer via activating Wnt signaling pathway. Mol Cancer. 2021;20:156. 34856993 10.1186/s12943-021-01469-6 PMC8638142

[18] Yang R Liu Z Cao H. LINC01089, suppressed by YY1, inhibits lung cancer progression by targeting miR-301b-3p/HPDG axis. Cell Biol Toxicol. 2021:10.1007/s10565-021-09643-8. 10.1007/s10565-021-09643-8 34561789

[19] Britt KL Cuzick J Phillips KA. Key steps for effective breast cancer prevention. Nat Rev Cancer. 2020;20:417–36. 32528185 10.1038/s41568-020-0266-x

[20] Shen X Zhong J Yu P. YY1-regulated LINC00152 promotes triple negative breast cancer progression by affecting on stability of PTEN protein. Biochem Biophys Res Commun. 2019;509:448–54. 30594392 10.1016/j.bbrc.2018.12.074

[21] Shen B Li Y Ye Q. YY1-mediated long non-coding RNA Kcnq1ot1 promotes the tumor progression by regulating PTEN via DNMT1 in triple negative breast cancer. Cancer Gene Ther. 2021;28:1099–112. 33323961 10.1038/s41417-020-00254-9

[22] Csolle MP Ooms LM Papa A. PTEN and other PtdIns (3,4,5) P (3) lipid phosphatases in breast cancer. Int J Mol Sci. 2020;21:9189. 33276499 10.3390/ijms21239189 PMC7730566

[23] Qiao K Ning S Wan L. LINC00673 is activated by YY1 and promotes the proliferation of breast cancer cells via the miR-515-5p/MARK4/Hippo signaling pathway. J Exp Clin Cancer Res. 2019;38:418. 31623640 10.1186/s13046-019-1421-7 PMC6796384

[24] Yang Z Ding H Pan Z. YY1-inudced activation of lncRNA DUXAP8 promotes proliferation and suppresses apoptosis of triple negative breast cancer cells through upregulating SAPCD2. Cancer Biol Ther. 2021;22:216–24. 33683171 10.1080/15384047.2021.1881201 PMC8043174

[25] Reifenberger G Wirsching HG Knobbe-Thomsen CB. Advances in the molecular genetics of gliomas - implications for classification and therapy. Nat Rev Clin Oncol. 2017;14:434–52. 28031556 10.1038/nrclinonc.2016.204

[26] Mazor G Smirnov D Ben David H. TP73-AS1 is induced by YY1 during TMZ treatment and highly expressed in the aging brain. Aging. 2021;13:14843–61. 34115613 10.18632/aging.203182 PMC8221307

[27] Chen L Gong X Huang M. YY1-Activated long noncoding RNA SNHG5 promotes glioblastoma cell proliferation through p38/MAPK signaling pathway. Cancer Biother Radiopharm. 2019;34:589–96. 31657621 10.1089/cbr.2019.2779

[28] Li F Shen ZZ Xiao CM. YY1-mediated up-regulation of lncRNA LINC00466 facilitates glioma progression via miR-508/CHEK1. J Gene Med. 2021;23:e3287. 33037684 10.1002/jgm.3287

[29] Neizer-Ashun F Bhattacharya R. Reality CHEK: understanding the biology and clinical potential of CHK1. Cancer Lett. 2021;497:202–11. 32991949 10.1016/j.canlet.2020.09.016

[30] Li H Li T Huang D. Long noncoding RNA SNHG17 induced by YY1 facilitates the glioma progression through targeting miR-506-3p/CTNNB1 axis to activate Wnt/*β*-catenin signaling pathway. Cancer Cell Int. 2020;20:29. 32009853 10.1186/s12935-019-1088-3 PMC6988207

[31] Liu P Zhao P Li B. LncRNA PCAT6 regulated by YY1 accelerates the progression of glioblastoma via miR-513/IGF2BP1. Neurochem Res. 2020;45:2894–902. 32990800 10.1007/s11064-020-03138-4

[32] Huang X Zhang H Guo X. Insulin-like growth factor 2 mRNA-binding protein 1 (IGF2BP1) in cancer. J Hematol Oncol. 2018;11:88. 29954406 10.1186/s13045-018-0628-y PMC6025799

[33] Kanth P Inadomi JM. Screening and prevention of colorectal cancer. BMJ. 2021;374:n1855. 34526356 10.1136/bmj.n1855

[34] Ye Y Gu B Wang Y. YY1-Induced upregulation of long noncoding RNA ARAP1-AS1 promotes cell migration and invasion in colorectal cancer through the Wnt/*β*-Catenin signaling pathway. Cancer Biother Radiopharm. 2019;34:519–28. 31173500 10.1089/cbr.2018.2745

[35] Wang Y Wu S Yang X. Association between polymorphism in the promoter region of lncRNA GAS5 and the risk of colorectal cancer. Biosci Rep. 2019;39:BSR20190091. 30902880 10.1042/BSR20190091 PMC6465203

[36] Ni W Yao S Zhou Y. Long noncoding RNA GAS5 inhibits progression of colorectal cancer by interacting with and triggering YAP phosphorylation and degradation and is negatively regulated by the m (6)A reader YTHDF3. Mol Cancer. 2019;18:143. 31619268 10.1186/s12943-019-1079-y PMC6794841

[37] Tian JB Cao L Dong GL. Long noncoding RNA DDX11-AS1 induced by YY1 accelerates colorectal cancer progression through targeting miR-873/CLDN7 axis. Eur Rev Med Pharmacol Sci. 2019;23:5714–29. 31298324 10.26355/eurrev_201907_18309

[38] Gu J Dong L Wang Y. LINC01224 promotes colorectal cancer progression through targeting miR-485-5p/MYO6 axis. World J Surg Oncol. 2021;19:281. 34535152 10.1186/s12957-021-02389-x PMC8449439

[39] Yang JD Hainaut P Gores GJ. A global view of hepatocellular carcinoma: trends, risk, prevention and management. Nat Rev Gastroenterol Hepatol. 2019;16:589–604. 31439937 10.1038/s41575-019-0186-y PMC6813818

[40] Wu Y Zhao Y Huan L. An LTR retrotransposon-derived long noncoding RNA lncMER52A promotes hepatocellular carcinoma progression by binding p120-catenin. Cancer Res. 2020;80:976–87. 31874857 10.1158/0008-5472.CAN-19-2115

[41] Song H Liu Y Li X. Long noncoding RNA CASC11 promotes hepatocarcinogenesis and HCC progression through EIF4A3-mediated E2F1 activation. Clin Transl Med. 2020;10:e220. 33252856 10.1002/ctm2.220 PMC7643871

[42] Cheng T Yao Y Zhang S. LINC00205, a YY1-modulated lncRNA, serves as a sponge for miR-26a-5p facilitating the proliferation of hepatocellular carcinoma cells by elevating CDK6. Eur Rev Med Pharmacol Sci. 2021;25:6208–19. 34730201 10.26355/eurrev_202110_26991

[43] Morrison AH Byrne KT Vonderheide RH. Immunotherapy and prevention of pancreatic cancer. Trends Cancer. 2018;4:418–28. 29860986 10.1016/j.trecan.2018.04.001 PMC6028935

[44] Huang X Pan L Zuo Z. LINC00842 inactivates transcription co-regulator PGC-1*α* to promote pancreatic cancer malignancy through metabolic remodelling. Nat Commun. 2021;12:3830. 34158490 10.1038/s41467-021-23904-4 PMC8219694

[45] Hu Y Tang J Xu F. A reciprocal feedback between N6-methyladenosine reader YTHDF3 and lncRNA DICER1-AS1 promotes glycolysis of pancreatic cancer through inhibiting maturation of miR-5586-5p. J Exp Clin Cancer Res. 2022;41:69. 35183226 10.1186/s13046-022-02285-6 PMC8857805

[46] Jiang S Zhang Q Li J. New sights into long Non-Coding RNA LINC01133 in cancer. Front Oncol. 2022;12:908162. 35747817 10.3389/fonc.2022.908162 PMC9209730

[47] Yang X Wang L Zhou F. Yin Yang 1-induced activation of LINC01133 facilitates the progression of pancreatic cancer by sponging miR-199b-5p to upregulate myelin regulatory factor expression. Bioengineered. 2022;13:13352–65. 35659199 10.1080/21655979.2022.2038900 PMC9275991

[48] Lheureux S Gourley C Vergote I. Epithelial ovarian cancer. Lancet. 2019;393:1240–53. 30910306 10.1016/S0140-6736(18)32552-2

[49] Yang H Zhang X Zhu L. YY1-Induced lncRNA PART1 enhanced resistance of ovarian cancer cells to cisplatin by regulating miR-512-3p/CHRAC1 axis. DNA Cell Biol. 2021;40:821–32. 34030482 10.1089/dna.2021.0059

[50] Cohen PA Jhingran A Oaknin A. Cervical cancer. Lancet. 2019;393:169–82. 30638582 10.1016/S0140-6736(18)32470-X

[51] Liu H Xu J Yang Y. Oncogenic HPV promotes the expression of the long noncoding RNA lnc-FANCI-2 through E7 and YY1. Proc Natl Acad Sci USA. 2021;118:e2014195118. 33436409 10.1073/pnas.2014195118 PMC7826414

[52] Kajiyama H Suzuki S Yoshihara M. Endometriosis and cancer. Free Radic Biol Med. 2019;133:186–92. 30562557 10.1016/j.freeradbiomed.2018.12.015

[53] Hu W Xie Q Xu Y. Integrated bioinformatics analysis reveals function and regulatory network of miR-200b-3p in endometriosis. Biomed Res Int. 2020;2020:3962953. 32802844 10.1155/2020/3962953 PMC7414375

[54] Cao W Lee H Wu W. Multi-faceted epigenetic dysregulation of gene expression promotes esophageal squamous cell carcinoma. Nat Commun. 2020;11:3675. 32699215 10.1038/s41467-020-17227-z PMC7376194

[55] Cheng J Yang Q Han X. Yin Yang 1-stimulated long noncoding RNA bladder cancer-associated transcript 1 upregulation facilitates esophageal carcinoma progression via the microRNA-5590-3p/programmed cell death-ligand 1 pathway. Bioengineered. 2022;13:10244–57. 35435118 10.1080/21655979.2022.2061303 PMC9161860

[56] Li Y Li T Yang Y. YY1-induced upregulation of FOXP4-AS1 and FOXP4 promote the proliferation of esophageal squamous cell carcinoma cells. Cell Biol Int. 2020;44:1447–57. 32159250 10.1002/cbin.11338

[57] Wei X Liu F Jiang X. YY1 promotes telomerase activity and laryngeal squamous cell carcinoma progression through impairment of GAS5-Mediated p53 stability. Front Oncol. 2021;11:692405. 34497757 10.3389/fonc.2021.692405 PMC8421032

[58] Chen X Xu W Ma Z. TTN-AS1 accelerates the growth and migration of nasopharyngeal carcinoma cells via targeting miR-876-5p/NETO2. Mol Ther Oncolytics. 2022;24:535–46. 35229031 10.1016/j.omto.2021.11.009 PMC8851086

[59] Sun D Zhao Y Wang W. PCAT1 induced by transcription factor YY1 promotes cholangiocarcinoma proliferation, migration and invasion by sponging miR-216a-3p to up-regulate oncogene BCL3. Biol Chem. 2021;402:207–19. 33544468 10.1515/hsz-2020-0276

[60] Li J Guan C Hu Z. Yin Yang 1-induced LINC00667 up-regulates pyruvate dehydrogenase kinase 1 to promote proliferation, migration and invasion of cholangiocarcinoma cells by sponging miR-200c-3p. Hum Cell. 2021;34:187–200. 33040228 10.1007/s13577-020-00448-1

[61] Zhang T Beeharry MK Wang Z. YY1-modulated long non-coding RNA SNHG12 promotes gastric cancer metastasis by activating the miR-218-5p/YWHAZ axis. Int J Biol Sci. 2021;17:1629–43. 33994849 10.7150/ijbs.58921 PMC8120461

[62] Zhang M Xu Y Yin S. YY1-induced long non-coding RNA PSMA3 antisense RNA 1 functions as a competing endogenous RNA for microRNA 214-5p to expedite the viability and restrict the apoptosis of bladder cancer cells via regulating programmed cell death-ligand 1. Bioengineered. 2021;12:9150–61. 34720049 10.1080/21655979.2021.1994907 PMC8809964

[63] Shan B Qu S Lv S. YY1-induced long non-coding RNA small nucleolar RNA host gene 8 promotes the tumorigenesis of melanoma via the microRNA-656-3p/SERPINE1 mRNA binding protein 1 axis. Bioengineered. 2022;13:4832–43. 35156513 10.1080/21655979.2022.2034586 PMC8973976

[64] Hua JT Ahmed M Guo H. Risk SNP-Mediated promoter-enhancer switching drives prostate cancer through lncRNA PCAT19. Cell. 2018;174:564–575.e18. 30033362 10.1016/j.cell.2018.06.014

[65] Li ZJ Cheng J Song Y. LncRNA SNHG5 upregulation induced by YY1 contributes to angiogenesis via miR-26b/CTGF/VEGFA axis in acute myelogenous leukemia. Lab Invest. 2021;101:341–52. 33318617 10.1038/s41374-020-00519-9

[66] Yao W Yan Q Du X. TNK2-AS1 upregulated by YY1 boosts the course of osteosarcoma through targeting miR-4319/WDR1. Cancer Sci. 2021;112:893–905. 33164271 10.1111/cas.14727 PMC7893995

[67] Gu J Dai B Shi X. lncRNA HCG11 suppresses human osteosarcoma growth through upregulating p27 Kip1. Aging. 2021;13:21743–57. 34518440 10.18632/aging.203517 PMC8457558

[68] Su H Liu L Zhang Y. Long noncoding RNA NPCCAT1 promotes nasopharyngeal carcinoma progression via upregulating YY1. Biochimie. 2019;157:184–94. 30481541 10.1016/j.biochi.2018.11.014

[69] Yu Q Zhang W Zhou X. Regulation of lnc-TLCD2-1 on radiation sensitivity of colorectal cancer and comprehensive analysis of its mechanism. Front Oncol. 2021;11:714159. 34336703 10.3389/fonc.2021.714159 PMC8320535

[70] Dong X Xu X Guan Y. LncRNA LINC00899 promotes progression of acute myeloid leukaemia by modulating miR-744-3p/YY1 signalling. Cell Biochem Funct. 2020;38:955–64. 32157707 10.1002/cbf.3521

[71] Zhang J Li N Fu J. Long noncoding RNA HOTAIR promotes medulloblastoma growth, migration and invasion by sponging miR-1/miR-206 and targeting YY1. Biomed Pharmacother. 2020;124:109887. 31986414 10.1016/j.biopha.2020.109887

[72] Zhong Y Lin H Li Q. Downregulation of long non-coding RNA GACAT1 suppresses proliferation and induces apoptosis of NSCLC cells by sponging microRNA-422a. Int J Mol Med. 2021;47:659–67. 33416153 10.3892/ijmm.2020.4826 PMC7797425

[73] Rong D Dong Q Qu H. m (6)A-induced LINC00958 promotes breast cancer tumorigenesis via the miR-378a-3p/YY1 axis. Cell Death Discovery. 2021;7:27. 33531456 10.1038/s41420-020-00382-z PMC7854648

[74] Wang N Duan H Zhang C. The LINC01186 suppresses cell proliferation and invasion ability in papillary thyroid carcinoma. Oncol Lett. 2018;16:5639–44. 30344719 10.3892/ol.2018.9349 PMC6176258

[75] Fernando TR Contreras JR Zampini M. The lncRNA CASC15 regulates SOX4 expression in RUNX1-rearranged acute leukemia. Mol Cancer. 2017;16:126. 28724437 10.1186/s12943-017-0692-x PMC5517805

[76] Xuan W Zhou C You G. LncRNA LINC00668 promotes cell proliferation, migration, invasion ability and EMT process in hepatocellular carcinoma by targeting miR-532-5p/YY1 axis. Biosci Rep. 2020;40:BSR20192697. 32249890 10.1042/BSR20192697 PMC7214398

[77] Yang P Li J Peng C. TCONS_00012883 promotes proliferation and metastasis via DDX3/YY1/MMP1/PI3K-AKT axis in colorectal cancer. Clin Transl Med. 2020;10:e211. 33135346 10.1002/ctm2.211 PMC7568852

[78] Wu S Zhang L Deng J. A novel micropeptide encoded by Y-Linked LINC00278 links cigarette smoking and AR signaling in male esophageal squamous cell carcinoma. Cancer Res. 2020;80:2790–803. 32169859 10.1158/0008-5472.CAN-19-3440

[79] Xu P Xiao H Yang Q. The USP21/YY1/SNHG16 axis contributes to tumor proliferation, migration, and invasion of non-small-cell lung cancer. Exp Mol Med. 2020;52:41–55. 31956270 10.1038/s12276-019-0356-6 PMC7000404

[80] Luo ZF Peng Y Liu FH. Long noncoding RNA SNHG14 promotes malignancy of prostate cancer by regulating with miR-5590-3p/YY1 axis. Eur Rev Med Pharmacol Sci. 2020;24:4697–709. 32432733 10.26355/eurrev_202005_21158

[81] Yu J Wang F Zhang J. LINC00667/miR-449b-5p/YY1 axis promotes cell proliferation and migration in colorectal cancer. Cancer Cell Int. 2020;20:322. 32694944 10.1186/s12935-020-01377-7 PMC7368754

[82] Liu J Zhan Y Wang J. Long noncoding RNA LINC01578 drives colon cancer metastasis through a positive feedback loop with the NF-*κ*B/YY1 axis. Mol Oncol. 2020;14:3211–33. 33040438 10.1002/1878-0261.12819 PMC7718957

[83] Wang Y Dou L Qin Y. OIP5-AS1 contributes to tumorigenesis in hepatocellular carcinoma by miR-300/YY1-activated WNT pathway. Cancer Cell Int. 2020;20:440. 32943988 10.1186/s12935-020-01467-6 PMC7487829

[84] Guo T Liu D Peng S. A positive feedback loop of lncRNA MIR31HG-miR-361-3p -YY1 accelerates colorectal cancer progression through modulating proliferation, angiogenesis, and glycolysis. Front Oncol. 2021;11:684984. 34485123 10.3389/fonc.2021.684984 PMC8416113

[85] Rong Z Wang Z Wang X. Molecular interplay between linc01134 and YY1 dictates hepatocellular carcinoma progression. J Exp Clin Cancer Res. 2020;39:61. 32272940 10.1186/s13046-020-01551-9 PMC7146959

[86] Feng Z Ye Z Xie J. Study on the mechanism of LOXL1-AS1/miR-3614-5p/YY1 signal axis in the malignant phenotype regulation of hepatocellular carcinoma. Biology Direct. 2021;16:24. 34863279 10.1186/s13062-021-00312-8 PMC8645132

[87] Zhou X Zeng B Li Y. LINC02532 contributes to radiosensitivity in clear cell renal cell carcinoma through the miR-654-5p/YY1 axis. Molecules. 2021;26:7040. 34834139 10.3390/molecules26227040 PMC8625588

[88] Xu L Wu Q Yan H. Long noncoding RNA KB-1460A1.5 inhibits glioma tumorigenesis via miR-130a-3p/TSC1/mTOR/YY1 feedback loop. Cancer Lett. 2022;525:33–45. 34728310 10.1016/j.canlet.2021.10.033

